# Homœopathy in Veterinary Practice

**Published:** 1882-01

**Authors:** 


### EDITORIAL DEPARTMENT.

### HOMŒOPATHY IN VETERINARY PRACTICE.

THERE are in this city and in Brooklyn several veterinarians who profess to practice in accordance with the homœopathic dogma, that "like cures like." A small minority of veterinarians throughout the country do the same. These persons, so far as we can learn, may be divided into two classes: those who are too lazy or ignorant to practice any other way, and those who from experience have concluded that the domestic animals do not need much medicine of any kind; and they think perhaps that homœopathic medicines are harmless to the horse, yet reassuring to the owner.

It would seem as though in veterinary practice there might be some positive facts obtained either for or against homœopathy. Veterinarians have none of the bitter personal feelings on the subject which are possessed by so many human practitioners. It is and should be a matter of indifference as to the standing of a veterinarian what drugs he used, provided he uses them intelligently and honestly. Furthermore, it is claimed in human medicine that the effects of homœopathic drugs are largely dependent on mental influence. There can be none of this in veterinary practice.

We have made a number of inquiries, with the hope of learning something positive regarding the practical results of homœopathic drugs, when given to the lower animals. So far as can be ascertained, however, there is no homœopathy at all in their mode of administration, and there are no certain therapeutical results of any kind. A number of mother tinctures are given emperically. There have been no "provings," as indeed there could not be, to any extent. These "mother tinctures" (which are nearly as strong as fluid extracts) are given often, according to the indications furnished by physiological science. Thus, nux vomica is given for constipation; aconite and verdrum viride for fevers; bryonia in lung troubles, etc.; cantharides is given in kidney diseases; arsenic and camomile in diarrhœa and colic, etc. The practice, in this neighborhood at least, seems to be one quite different from that laid down in a certain homœopathic veterinary manual which has been published for the guidance of the transcendentally inclined.

In fact, we are obliged to conclude that there is nothing to be learned out of veterinary homœopathy, unless it is that animals get well oftener than one would suppose, when left alone. An ignorant man had better practice homœopathy, because he can do no harm except in a negative way. An intelligent man will not practice it, unless he is a nihilist in therapeutics. And we believe that persons who are nihilists in therapeutics are those who do not understand how to give remedies.

It is not impossible that among the drugs used by homœopaths there are some which might be wisely appropriated by those of the regular school. This possibility should be borne in mind. Furthermore, there is no doubt that almost every remedy has a double action, according to the amount given. This, too, is a fact that homœopaths should not be allowed to monopolize.

